# Psychometric Testing of HeLD-14 in a Colombian Geriatric Population

**DOI:** 10.1155/2024/5570671

**Published:** 2024-02-07

**Authors:** Ana Cristina Mafla, Mauricio Herrera-López, Carmen Gallardo-Pino, Falk Schwendicke

**Affiliations:** ^1^Escuela Internacional de Doctorado, Universidad Rey Juan Carlos, Madrid, Spain; ^2^School of Dentistry, Universidad Cooperativa de Colombia, Pasto, Colombia; ^3^Department of Psychology, Universidad de Nariño, Pasto, Colombia; ^4^Departamento de Especialidades Médicas y Salud Pública, Facultad Ciencias de la Salud, Universidad Rey Juan Carlos, Madrid, Spain; ^5^Department of Oral Diagnostics, Digital Health and Health Services Research, Charité Universitätsmedizin Berlin, Berlin, Germany

## Abstract

**Introduction:**

The objective of this study was to test the validity and reliability of the Colombian version of the Health Literacy in Dentistry (HeLD-14) in older adults.

**Materials and Methods:**

A translation and validation study of HeLD-14 was conducted on 384 non-institutionalized older adults attending the Dental Clinic at Universidad Cooperativa from Pasto, Colombia. A cross-cultural adaptation of a multidimensional HeLD-14 was completed, and the psychometric properties of this scale were evaluated through a cross-validation method using an exploratory factor analysis (EFA) and a confirmatory factor analysis (CFA). Internal consistency was measured with Cronbach's alpha (*α*) and Omega's McDonald (*ɷ*). The statistical significance was set at *P* < 0.05.

**Results:**

The EFA demonstrated that a single-factor structure with 11 items explained a cumulative 59.86% of the overall variance. The CFA confirmed that goodness of fit indices of this questionnaire had optimal adequateness (*χ*^2^_S-B_ = 109.047; *χ*^2^_S-B_/(44) = 2.478, *P*=0.001; non-normed fit index = 0.901; comparative fit index = 0.908; root mean square error of approximation = 0.079 (90% CI (0.075, 0.083)); standardized root mean residual = 0.080). The coefficients indicated a high internal consistency for the total scale (*α* = 0.94; *ɷ* = 0.96).

**Conclusion:**

The developed adaptation of HeLD-14 for the Colombian population, HeLD-Col, is a unidimensional, reliable, and valid instrument to assess oral health literacy in older adults in Colombia.

## 1. Introduction

Oral health literacy (OHL) is known as “the degree to which individuals have the capacity to obtain, process, and understand basic health information and services needed to make appropriate oral health decisions” [1, 2]. OHL encompasses skills needed to use health information effectively [3]. A limited OHL has been associated with a higher prevalence of periodontal disease [4, 5], more missing teeth [6], and low utilization of oral healthcare [7].

Several tools to measure OHL, such as Rapid Estimate of Adult Literacy in Dentistry, Test of Functional Health Literacy in Dentistry, Oral Health Literacy Instrument, Oral Health Literacy Adults Questionnaire, and Health Literacy in Dentistry (HeLD-29, HeLD-14), among others have been developed [8]. However, most rely on measuring reading skills relative to oral health content and the identification of dental definitions instead of measuring the construct.

The Health Literacy in Dentistry (HeLD-29) [9] is an instrument that has seven dimensions such as communication, understanding, receptivity, utilization, support, finance, and access. HeLD-14 [10] is a short form of HeLD-29, with response options graded on a 5-point Likert–scale. HeLD-14 has been translated and validated into Brazilian–Portuguese [11, 12] and has been found to show good reliability and validity. Few studies, though, have evaluated its psychometric properties in older adults, and generally, the quality of validating translated OHL instruments has been found to be limited [13].

Measuring OHL is relevant for research and clinical practice, e.g., as a screening tool, especially for older adults who frequently suffer from oral health conditions that affecting their quality of life [14]. Validating translated OHL instruments like HeLD-14 in specific social contexts and populations is relevant, which is why the objective of the present study was to test the validity and reliability of HeLD-14 or its specific adaptation in Colombian older adults.

## 2. Materials and Methods

### 2.1. Study, Sample, and Settings

A cross-sectional survey was carried out among older adults attending the Dental Clinic at Universidad Cooperativa de Colombia, from Pasto, Colombia, between September 2021 and April 2022. A convenience sample (non-probability sampling) of 384 was consecutively approached in the mentioned period of time. Older adults ≥65 years old who were in sufficiently good physical and mental health to coherently answer a questionnaire were included. After the evaluation, eight participants who presented back-pain, urinary incontinence, and dental anxiety or had no time to answer the questionnaire were excluded. Pre-interview preparation meetings were carried out. They consisted in reviewing the questionnaire, discussing about the key questions' participants may ask to the interviewers, and preparing the focus and alignment of the answers (e.g., if someone did not use the word “folleto,” alternative words such as “volante,” “cartilla,” or “catálogo” would be used). A face-to-face interview in the dental unit at the clinic was conducted by two calibrated researchers to obtain information about sociodemographic characteristics and OHL, taking between 10 and 20 min.

### 2.2. Instrument

As mentioned, HeLD-14 [10] is a short version of HeLD-29 [9] with 14 items, which are rated on a five-point Likert scale from 0 = “unable to do” to 4 = “without any difficulty.” The total score ranges between 0 and 56. A higher score denotes a higher OHL. The original version was utilized to maintain the structure and warrant the theoretical robustness of the construct; moreover, items such as “*Are you able to pay for dental medication*” (found in this version) was more suitable than “*Are you able to pay to see a dentist*” in our socioeconomic context. As mentioned before, this questionnaire has been validated in Australian adults [15], Brazilian adults [11] and older adults [12], in Chinese adults [16] through an adapted version, and in Thai adults with physical disabilities using an exploratory factor analysis (EFA) [17]. Permission to use the questionnaire was granted by the author.

To obtain the Spanish version of HeLD-14, we completed a cross-cultural adaptation process [18] that included translation, synthesis, back translation, expert committee review, pretesting, and final testing, as described in the following sections.

#### 2.2.1. Translation

The HeLD-14, Spanish version, included two independent forward translations from English to Spanish. A bilingual professional in dentistry (TR-1) and a bilingual professional in psychology (TR-2) reported their translated versions of the HeLD-14, as well as response options and instructions of the questionnaire. Similarly, in this step, comments such as challenging sentences or ambiguous wording in this questionnaire were expressed.

#### 2.2.2. Synthesis

The HeLD-14 Spanish versions from the first translator (TR-1) and second translator (TR-2) were analyzed, discussed, and synthesized by the researchers. Additionally, a Spanish/English professor (TR-3) arbitrated a discussion between the TR-1 and TR-2. To develop a unified Spanish version of the HeLD-14, the consensus of the three translators (TR-123) was made.

#### 2.2.3. Back Translation

Two translators, a native English professor (BT-1) and a bilingual health professional (linked to the American health system) (BT-2), who did not know the original questionnaire of HeLD-14, back-translated the Spanish version into English. The researchers consolidated the back-translated versions of the HeLD-14 questionnaire and compared them to the original version to determine the validity of the translation process (Table 1).

#### 2.2.4. Expert Committee Review

The committee consisted of dental and psychology professionals and translators who consolidated the different versions of the questionnaire to ensure equivalence between the original version of the HeLD-14 and the new Spanish version.

#### 2.2.5. Pretesting

The translated questionnaire was pretested in a sample of 20 older adults from the Dental Clinic at Universidad Cooperativa de Colombia, from Pasto, Colombia, using standard interview techniques.

#### 2.2.6. Testing

After the pre-testing process, the researchers tested the final Spanish-translated version of HeLD-14. A face-to-face interview about sociodemographic characteristics, including variables such as age [19], sex [20], socioeconomic status (SES) [21], permanent residency, health insurance, education, occupation, and OHL, was conducted. This interview lasted between 10 and 20 min.

## 3. Analytic Strategy

### 3.1. Preliminary Analysis

Descriptive analyses were performed to determine the distribution of sociodemographic characteristics of the sample and estimate the HeLD-14 items scale measures (frequencies, means, standard deviations, skewness, kurtosis, and inter-item correlations). Then, Mardia's coefficient was calculated for testing the multivariate normality of the data using the “R” statistical program [22]. For construct validity, the sample was randomly split into two equivalent samples to conduct factor analyses utilizing a cross-validation method. The first subsample was used to perform an EFA, and the second subsample for a confirmatory factor analysis (CFA). These procedures sought to make sequential use of these two analyses to explore the distribution of the items and confirm the theoretical model of the measurement scale [23, 24].

### 3.2. EFA

The EFA was calculated with the Factor 9.2 program [25] using the principal-axis factor analysis conducted with a direct Oblimin rotation method. In this analysis, the sampling adequacy measures such as the Kaiser–Meyer–Olkin (KMO) test (<1), Bartlett's sphericity, communality values, item saturations, and factor loadings were obtained in a distribution-free of the items (according to the matrix configuration), and the total explained variance. The final factor structure was obtained using the following criteria: retained items with factor loadings ≥0.30, retained factors that had ≥3 items, and internal consistency of multi-item ≥0.70.

### 3.3. CFA

Finally, to verify the factor structure obtained in the EFA, a CFA was performed using the unweighted least squares estimation method. As we used ordinal variables, our data were not multivariate normally distributed [26, 27], and the inter-item correlations were high, polychoric correlations were estimated. Additionally, Satorra and Bentler [28] *χ*^2^ (*χ*^2^_S-B_) *χ*^2^_S-B_ /degrees of freedom (*χ*^2^_S-B_ /df) (≤3), comparative fit index (CFI) (≥0.90), non-normed fit index (NNFI) (≥0.90), root mean square error of approximation (RMSEA) (≤0.08) and the standardized root mean residual (SRMR) (≤0.08) [29] were used to evaluate the model fit. These indices were generated by the EQS-6.2 program [30].

### 3.4. Reliability

Finally, to test the internal consistency of the scale, Omega's McDonald (*ɷ*) [31] and Cronbach's alpha (*α*) were estimated (values <0.70 indicated unacceptable levels of reliability) using the Factor 9.2 program [25]. The significance was set at *P* < 0.05.

## 4. Results

### 4.1. Study Participants

The sample consisted of 224 male (58.3%) and 160 female (41.7%) individuals. Their age ranged between 65 and 89 years (mean = 70.56; standard deviation, SD = 4.98 years). Overall, 285 (74.2%) older adults belonged to a low SES, 60 (15.6%) to middle SES, and 39 (10.2%) to high SES. A total of 233 (60.7%) lived in Pasto city (capital) and 151 (39.3%) in other places. Overall, 382 (99.5%) participants reported having public health insurance; among them, 232 (60.5%) had subsidized insurance, 90 (23.4%) had contributory insurance, and 60 (15.6%) had a private insurance. Regarding education, 299 (77.9%) had completed primary and secondary school, 44 (11.5%) had a 2-year college degree, and 41 (10.7%) had university studies. Overall, 29 (7.6%) participants were retired, 90 (23.4%) were pensioners, and 265 (69.0%) were still working in different kinds of jobs.

### 4.2. Psychometric Properties

Table 2 displays the descriptive analysis for the scale as well as for items. Mardia's analysis demonstrated a skewness of 35.50 *P* < 0.001 and kurtosis of 240.74, *P* < 0.001, indicating that the data were not multivariate normally distributed.

An EFA was initially conducted to validate the construct of the Colombian version of the HeLD-14 scale. The results showed a substantial adequacy of the KMO test (0.918) and a significant Bartlett sphericity test (*χ*^2^ = 3537.518; *df* = 55; *P*  < 0.001). The communalities were adequate, ranging from 0.298 (Item 1) to 0.761 (Item 8). Then, a factorial distribution was tested in a distribution-free which exhibited that after removing Items 2, 3, and 6 because of weak factor loading (≤0.30) or loading similarly on two factors, the retained 11 items loaded in a single factor with an explained variance of 59.86% (Table 3).

**Figure 1:**
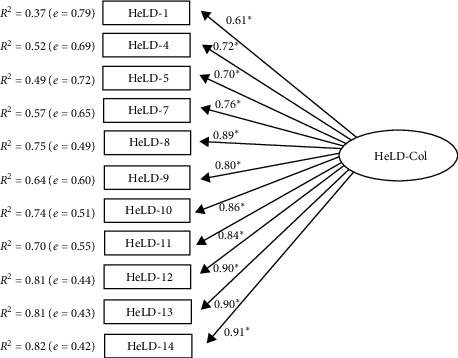
Confirmatory factor analysis for HeLD Colombian (Col) version ^ ^*∗*^^(*P* < 0.05).

As a unidimensional factor structure was obtained from the EFA, a single-factor structure was tested using a CFA. The goodness of fit indices of the HeLD-Col questionnaire confirmed optimal adequateness (*χ*^2^_S-B_ = 109.047; *χ*^2^_S-B_/(44) = 2.478, *P*=0.001; NNFI = 0.901; CFI = 0.908; RMSEA = 0.079 (90% CI (0.075, 0.083)); SRMR = 0.080). Moreover, lambda (*λ*) values, determination coefficients (*R*^2^), and measurement errors (*e*) were also adequate (Figure 1). The observed results of EFA and CFA showed that the one-factor structure best fits the data of Colombian older adults. The coefficients indicated high internal consistency for the total scale (*α* = 0.94; *ɷ* = 0.96).

## 5. Discussion

There is a particular research interest in abilities that may control the disease's development, such as OHL. To extend this knowledge, we have tested the psychometric properties of an important tool such as the HeLD-14 questionnaire in the Colombian population.

The means and the standard deviations of the HeLD-Col items were displayed to establish scores in different samples of individuals. These values were similar and slightly higher compared to a previous study in the Brazilian older population [12]. Interestingly, questions such as “*Are you able to pay to see a dentist?”* (mean = 1.5, SD = 1.5) included in the controlled selection of the instrument [10] and “*Are you able to pay for dental medication?”* (mean = 2.0, SD = 1.4) had lower scores in Brazilian older adults, while in our scale the last question exhibited a higher score (mean = 3.02, SD = 0.73). Moreover, in our study, higher scores were observed in both “*Are you able to carry out dental instructions*?” (Item 13) and “*Are you able to use dentist advice*?” (Item 14) questions. It may reflect the confidence of older adults to perform the actions required to complete successful dental treatments, and this ability may be related to self-efficacy [32, 33]. Item 4, “*Are you able to read dental information brochures?”* had the lowest score. Generally, in our locality, there is few accessible information about dentistry in brochures in dental offices and dental clinics. Further, some participants may be illiterate, especially those living in rural areas [34] who did not know or read such as brochures.

The EFA identified one-factor structure, as one preliminary criterion was to retain factors that include at least three items in order to decrease the variability of the construct measurement. The removed questionnaire items were “*Are you able to make time for things good for dental health?”* (Item 2), “*Are you able to read written information, e.g., leaflets given to you by your dentist?”* (Item 3), and “*Are you able to ask for support to a dental appointment?”* (Item 6). Item 2, for example, could lead to confusion among responders because in developing countries, it is not about “to make time”; it may be more about “to have money to pay for a treatment.” Regarding Item 3, dentists usually do not give to patients written information about dentistry in the dental office or dental clinics; they frequently leave entertainment magazines in the waiting room; for this reason, this may be an unknown situation. Finally, Item 6 might not load as part of the OHL questionnaire structure because, in our health system, patients usually must call to get a dental appointment. As older adults are not familiar with answering machines, they prefer to go by themselves (being self-sufficient) to healthcare settings to get their own dental appointments.

Additionally, Item 1, “*Are you able to pay attention to dental health needs?”* and Item 8, “*Do you know how to get dentist's appointment?”* had the lowest and the highest factor loading, respectively, indicating the relevance of these items to explain the construct. Item 1 could load less because the dental needs may be more complicated to determine, as the signs and symptoms of some oral diseases may be difficult to detect at early stages, and even in severe conditions such as periodontal disease, and Item 8 could load more because it is related to the health system utilization, definitely more precise.

The CFA showed this unidimensional scale with 11 items converged to indicate good psychometric performance as a measure of OHL and demonstrate validity in the key indices of the CFA. The unidimensionality of an instrument is important to interpret a total score; for example, if the items of a scale do not represent the same underlying construct, the meaning of a total score is distorted, and theoretical imprecisions could be introduced [35]. The unidimensional measurement is very useful for clinical applications, as it ensures that only one measurement construct is assessed at a time point and can be compared for the same individual over time.

Internal consistency for HeLD-Col total scores exceeded (*α* = 0.94; *ɷ* = 0.96) an acceptability level for Cronbach's alpha (≥0.7) [36] as well as Omega's McDonald [37] (between 0.7 and 0.9). The studies of psychometric properties of HeLD-14 conducted in Australian indigenous, Brazilian adults, and Brazilian older adults individuals displayed good psychometric properties even using factors with two items [10–12] with values of Cronbach's *α* of 0.87 in Australian indigenous [10], 0.86–0.88 in Brazilian adults [11] and 0.89 in Brazilian older population [12], those a little lower than our Cronbach's *α* of 0.94.

The results observed in this article will be useful for investigators and clinicians. Researchers should be aware that there exists a new version and validated questionnaire to measure OHL, that could be utilized as a screening tool to explore these abilities in other countries. Clinicians will also have a new tool to detect OHL skills, not only in older adults but in the general population. It would allow dental professionals to enhance required patients' abilities to find, understand, or use health information and services. This learning may help patients to prioritize decisions and actions for improving their oral health, especially in low-income people and countries where this type of care is imperative.

## 6. Conclusions

The HeLD-Col is a unidimensional, reliable, and valid instrument to assess OHL in older adults in the Colombian population. Future studies about psychometric properties of this unidimensional HeLD-Col are required to valid this scale equivalence across cultures.

## Figures and Tables

**Table 1:** Back-translation English–Spanish/Spanish/English of the HeLD-14 scale.

Instruction: Please complete this 10−20-min short survey to let us know your abilities to understand oral health information. All responses are recorded anonymously, so feel free to provide honest feedback		Instrucción: Complete esta breve encuesta de 10 a 20 min para dejarnos conocer sus habilidades para entender información de salud oral. Todas las respuestas serán registradas de forma anónima, así que siéntase libre de proporcionar una retroalimentación honesta.

Response options:		Opciones de respuesta:

0. Unable to do		0. Incapaz de hacerlo
1. Very difficult		1. Con mucha dificultad
2. With some difficulty		2. Con algo de dificultad
3. With little difficulty		3. Con poca dificultad
4. Without any difficulty		4. Sin dificultad

Item	Translation	HeLD-14 Questionnaire

HeLD-1 Q2	OAE	Are you able to pay attention to dental health needs?
	ST (TR-123)	¿Es capaz de prestar atención a sus necesidades odontológicas?
	BT-1	Are you able to pay attention to your dental needs?
	BT-2	Are you able to pay attention to your dental needs?

HeLD-2 Q3	OAE	Are you able to make time for things good for dental health?
	ST (TR-123)	¿Es capaz de dejar tiempo para mejorar su salud oral?
	BT-1	Are you able to make time to improve your oral health?
	BT-2	Are you able to make time to improve your oral health?

HeLD-3 Q7	OAE	Are you able to read written information, e.g., leaflets given to you by your dentist?
	ST (TR-123)	¿Es capaz de leer información escrita de odontología?
	BT-1	Are you able to read written information about dentistry?
	BT-2	Are you able to read written information concerning dentistry?
	Comment	The, e.g., was removed because leaflets are not usually given by dentists in the locality

HeLD-4 Q8	OAE	Are you able to read dental information brochures?
	ST (TR-123)	¿Es capaz de leer información odontológica de folletos?
	BT-1	Are you able to read dental information from brochures?
	BT-2	Are you able to read dental-related information from brochures?

HeLD-5 Q10	OAE	Are you able to take support to a dental appointment?
	ST (TR-123)	¿Necesita ir acompañado a una cita odontológica?
	BT-1	Do you need a companion to attend to a dental appointment?
	BT-2	Do you need company when attending a dentist's appointment?

HeLD-6 Q11	OAE	Are you able to ask for support to a dental appointment?
	ST (TR-123)	¿Es capaz de pedir apoyo para sacar una cita odontológica?
	BT-1	Are you able to ask for support to get a dental appointment?
	BT-2	Are you able to ask for help to schedule a dental appointment?

HeLD-7 Q15	OAE	Are you able to pay for dental medication?
	ST (TR-123)	¿Es capaz de pagar por medicamentos odontológicos? (por ejemplo, analgésicos, antibióticos, anti-inflamatorios, etc.)
	BT-1	Are you able to pay for dental medication? (for example, painkillers, antibiotics, anti-inflammatories)
	BT-2	Are you able to pay from your own pocket for a dental prescription? (for example, painkillers, antibiotics, anti-inflammatories)
	Comment	An example was added to clarify possible medications used in dentistry

HeLD-8 Q18	OAE	Do you know how to get dentist's appointment?
	ST (TR-123)	¿Usted sabe qué hacer para conseguir una cita odontológica?
	BT-1	Do you know how to get a dental appointment?
	BT-2	Do you know how to schedule a dental appointment?

HeLD-9 Q24	OAE	Are you able to get a second opinion?
	ST (TR-123)	¿Es capaz de obtener una segunda opinión?
	BT-1	Are you able to get a second opinion?
	BT-2	Are you able to ask for a second opinion?

HeLD-10 Q25	OAE	Are you able to look for a second opinion?
	ST (TR-123)	¿Es capaz de buscar una segunda opinión?
	BT-1	Are you able to look for a second opinion?
	BT-2	Are you able to look for a second opinion?

HeLD-11 Q26	OAE	Are you able to use information?
	ST (TR-123)	¿Es capaz de usar la información?
	BT-1	Are you able to use information?
	BT-2	Are you able to use information?

HeLD-12 Q27	OAE	Are you able to follow dental instructions?
	ST (TR-123)	¿Es capaz de seguir instrucciones odontológicas?
	BT-1	Are you able to follow dental instructions?
	BT-2	Are you able to follow dental instructions?

HeLD-13 Q28	OAE	Are you able to carry out dental instructions?
	ST (TR-123)	¿Es capaz de cumplir instrucciones odontológicas?
	BT-1	Are you able to comply dental instructions?
	BT-2	Are you able to follow dentists' orders?

HeLD-14 Q29	OAE	Are you able to use dentist advice?
	ST (TR-123)	¿Es capaz de seguir el consejo del odontólogo?
	BT-1	Are you able to follow dentist advice?
	BT-2	Are you able to follow dentist advice?

OAE, original: Australian English; ST, Spanish translation; TR-123, translation 123; BT-1, back-translation 1: American English; BT-2, back-translation 2: American English.

**Table 2:** Descriptive analysis of HeLD-Col items such as mean (*m*), standard deviation (SD), skewness, kurtosis, frequency (*F*), and percentage (%).

Items	*m*	SD	Skewness	Kurtosis	0 *F*/(%)	1 *F*/(%)	2 *F*/(%)	3 *F*/(%)	4 *F*/(%)
HeLD-1. Are you able to pay attention to dental health needs?	3.16	0.72	−0.582	0.144	0	8/2.1	51/13.3	197/51.3	128/33.3
HeLD-4. Are you able to read dental information brochures?	2.65	0.98	−0.345	−0.876	0	43/8.8	72/14.7	77/15.7	75/19.5
HeLD-5. Are you able to take support to a dental appointment?	2.87	0.81	0.130	−1.224	0	4/1.0	143/37.2	137/35.7	100/26.0
HeLD-7. Are you able to pay for dental medication?	3.02	0.73	−0.188	−0.624	0	4/1.0	86/22.4	194/50.5	100/26.0
HeLD-8. Do you know how to get dentist's appointment?	2.96	0.75	−0.050	−0.959	0	3/0.8	107/27.9	175/45.6	99/25.8
HeLD-9. Are you able to get a second opinion?	3.05	0.66	−0.327	0.504	1/0.3	1/0.3	66/17.2	225/58.6	91/23.7
HeLD-10. Are you able to look for a second opinion?	3.03	0.68	−0.142	−0.502	0	2/0.5	76/19.8	213/55.5	93/24.2
HeLD-11. Are you able to use information?	2.92	0.74	0.091	−1.080	0	1/0.3	119/31.0	174/45.3	90/23.4
HeLD-12. Are you able to follow dental instructions?	3.16	0.80	−0.334	−1.256	0	1/0.3	92/24.0	134/34.9	157/40.9
HeLD-13. Are you able to carry out dental instructions?	3.23	0.71	−0.368	−0.993	0	0	64/16.7	168/43.8	152/39.6
HeLD-14. Are you able to use dentist advice?	3.24	0.75	−0.415	−1.108	0	0	72/18.8	149/38.8	163/42.4

Response options: 0 = unable to do, 1 = very difficult, 2 = with some difficulty, 3 = with little difficulty, 4 = without any difficulty.

**Table 3:** Exploratory factor analysis for HeLD, Colombian (Col) version.

Item	Factor 1	*h* ^2^
HeLD-1	0.546	0.298
HeLD-4	0.711	0.505
HeLD-5	0.666	0.444
HeLD-7	0.734	0.539
HeLD-8	0.872	0.761
HeLD-9	0.790	0.625
HeLD-10	0.844	0.712
HeLD-11	0.796	0.633
HeLD-12	0.836	0.698
HeLD-13	0.821	0.674
HeLD-14	0.834	0.695
Total explained variance: 59.86%		

## Data Availability

The dataset used in the study is available from the corresponding author upon reasonable request.

## Additional Points

*Limitations*. There are some limitations in this study. First, our sample was not randomly distributed; for this reason, our findings might not be generalized to the entire population. Second, a social desirability bias might exist, as our data collection was based on a self-report questionnaire. Third, we could not evaluate other modern psychometric properties, such as item response theory, to explain the relationship between unobservable attributes and its performance.
